# Influences of Stress and Sex on the Paraventricular Thalamus: Implications for Motivated Behavior

**DOI:** 10.3389/fnbeh.2021.636203

**Published:** 2021-02-26

**Authors:** Sydney A. Rowson, Kristen E. Pleil

**Affiliations:** Department of Pharmacology, Weill Cornell Medicine, Cornell University, New York, NY, United States

**Keywords:** sex differences, paraventricular thalamus, stress, motivated behavior, HPA axis

## Abstract

The paraventricular nucleus of the thalamus (PVT) is a critical neural hub for the regulation of a variety of motivated behaviors, integrating stress and reward information from environmental stimuli to guide discrete behaviors *via* several limbic projections. Neurons in the PVT are activated by acute and chronic stressors, however several roles of the PVT in behavior modulation emerge only following repeated stress exposure, pointing to a role for hypothalamic pituitary adrenal (HPA) axis modulation of PVT function. Further, there may be a reciprocal relationship between the PVT and HPA axis in which chronic stress-induced recruitment of the PVT elicits an additional role for the PVT to regulate motivated behavior by modulating HPA physiology and thus the neuroendocrine response to stress itself. This complex interaction may make the PVT and its role in influencing motivated behavior particularly susceptible to chronic stress-induced plasticity in the PVT, especially in females who display increased susceptibility to stress-induced maladaptive behaviors associated with neuropsychiatric diseases. Though literature is describing the sex-specific effects of acute and chronic stress exposure on HPA axis activation and motivated behaviors, the impact of sex on the role of the PVT in modulating the behavioral and neuroendocrine response to stress is less well established. Here, we review what is currently known regarding the acute and chronic stress-induced activation and behavioral role of the PVT in male and female rodents. We further explore stress hormone and neuropeptide signaling mechanisms by which the HPA axis and PVT interact and discuss the implications for sex-dependent effects of chronic stress on the PVT’s role in motivated behaviors.

## Introduction

The paraventricular nucleus of the thalamus (PVT) is the most dorsal midline thalamic nucleus that extends across the anterior-posterior axis of the brain (Kirouac, 2015). The PVT sends projections to several brain regions involved in arousal, the stress response, and motivated behavior including the nucleus accumbens (NAc; Neumann et al., 2016; Zhu et al., 2016; Beas et al., 2018; Cheng et al., 2018), bed nucleus of the stria terminalis (BNST; Hua et al., 2018), central nucleus of the amygdala (CeA; Do-Monte et al., 2015; Penzo et al., 2015; Chen and Bi, 2019; Keyes et al., 2020), basolateral amygdala (BLA; Amir et al., 2019), and the prelimbic and infralimbic cortex (Gao et al., 2020). The PVT regulates both positively and negatively valenced motivated behaviors through its various limbic outputs, including natural reward and drug-seeking (Neumann et al., 2016; Zhu et al., 2016; Cheng et al., 2018), feeding (Cheng et al., 2018), approach-avoidance (Zhu et al., 2016; Cheng et al., 2018), and fear conditioning (Penzo et al., 2015), and these are potentially susceptible to the impact of stress. Also, there are distinct subpopulations of projection neurons and effects of acute and chronic stress across the anterior-posterior axis of the PVT (Bhatnagar and Dallman, 1998; Ver Hoeve et al., 2013; Gao et al., 2020), however, few studies have examined the relationship between these features (Beas et al., 2018; Gao et al., 2020).

The PVT is also activated by exposure to salient and stressful stimuli that activate the hypothalamic-pituitary-adrenal (HPA) axis and may be involved in integrating past experiences with present stimuli to guide adaptive behavior (Bhatnagar and Dallman, 1998; Bhatnagar et al., 2003; Choi and McNally, 2017; Beas et al., 2018; Zhu et al., 2018; Gao et al., 2020). As such, the PVT becomes activated upon each exposure to an acute stressor (Radley and Sawchenko, 2015), but it can develop a more robust role in behavior modulation across repeated stressor exposures (Bhatnagar et al., 2003). Therefore, this recruited role of the PVT indicates that the PVT is a site of plasticity in chronic stress-induced behavioral changes. This view is supported by converging evidence that stress hormones and neuropeptides, including glucocorticoids released directly as a result of activation of the HPA axis, contribute to stress-induced PVT plasticity.

Intriguingly, there is evidence that one stress-recruited role of the PVT in behavioral control is in modulating the HPA axis’s response to acute stress, thus affecting the way that the HPA axis can regulate PVT function in turn (Bhatnagar and Dallman, 1998; Bhatnagar et al., 2002). However, whether there is a reciprocal modulatory relationship between the PVT and HPA axis that emerges as a consequence of repeated stress is not yet fully clear. Understanding precisely how the PVT and HPA axis bidirectionally interact is important to understanding how the PVT can guide motivated behavior following stress exposure. Furthermore, given the well-established role of sex differences in HPA axis activity and responsivity to stress, the PVT’s role in regulating motivated behavior may be particularly susceptible to the sex-specific effects of stress. In this review, we discuss the literature detailing the interactions of sex, stress, and the HPA axis on the PVT and implications for its role in motivated behavior. This review will provide a framework for future studies to better clarify these complex interactions, providing essential information with implications for understanding sex differences in stress-related neuropsychiatric diseases.

## Acute Stress Effects on The Pvt, Hpa Axis, and Behavior

### Acute Stress and PVT Regulation of Motivated Behavior

While the roles of the PVT in motivated behaviors have been extensively reviewed elsewhere (Millan et al., 2017), the impact of and interaction between sex and stress on these roles is less well-examined. The PVT responds to both positively and negatively valenced salient stimuli and directly integrates information to modulate a variety of behaviors *via* limbic projections (Choi and McNally, 2017; Beas et al., 2018; Zhu et al., 2018; Gao et al., 2020). Some of the roles of the PVT are specifically toward signaling arousal (Gao et al., 2020) and salience (Zhu et al., 2018) that guide behavioral responses including fear (Do-Monte et al., 2015; Penzo et al., 2015), approach-avoidance (Zhu et al., 2016; Cheng et al., 2018), and drug-seeking behavior (Neumann et al., 2016; Zhu et al., 2016), all of which are sensitive to acute stress. Chemogenetic and optogenetic manipulation studies demonstrate that discrete anatomical outputs of the PVT are involved in these different behaviors. The projection from the PVT to the lateral division of the CeA is involved in fear conditioning (Penzo et al., 2015), and arousal is gated by populations of PVT neurons that project to the infralimbic cortex (Gao et al., 2020) and BNST (Hua et al., 2018). The PVT-NAc circuit has been implicated in drug-use behaviors like cocaine self-administration (Neumann et al., 2016) and aversion (including morphine withdrawal-induced aversion; Zhu et al., 2016), as well as feeding behavior in a novel environment (Cheng et al., 2018). Disrupting NAc-projecting PVT neurons reduces the acquisition of cocaine self-administration, indicating a role of the PVT-NAc circuit in early drug-seeking (Neumann et al., 2016).

The PVT participates in integrating multiple types of information to modulate behavior, including during motivational conflict, and the context and experimental conditions can impact the role of the PVT (Choi and McNally, 2017; Cheng et al., 2018; Choi et al., 2019). Under stressful conditions (food restriction) and in an anxiogenic context (novel environment), but not in the home cage, optogenetic activation of the anterior PVT (aPVT)-NAc circuit increases food consumption, indicating that this circuit promotes feeding during an approach-avoidance conflict (Cheng et al., 2018). BNST-projecting calretinin neurons in the PVT are activated by starvation to suppress sleep and promote arousal (Hua et al., 2018), and data from our group show that BNST-projecting PVT glutamate neurons are sufficient to reduce avoidance behavior in an anxiogenic context (elevated plus maze), an effect that may be due to feedforward inhibition of stress-responsive corticotropin-releasing factor (CRF) neurons in the BNST (Levine et al., 2020). Associative learning of salient stimuli rely on the PVT (Zhu et al., 2018), and the PVT is involved in balancing approach or avoidance behavior during situations of conflicting motivation following conditioning (Choi and McNally, 2017; Choi et al., 2019), discussed in “Chronic Stress and PVT Regulation of Motivated Behavior” section.

An additional layer of complexity in the role of the PVT in directing motivated behavior is that the PVT has functionally distinct populations of neurons across the anterior-posterior axis of the PVT. These different populations may be related to their differing circuit organization. For example, CeA projections regulating fear responses are primarily located in the posterior PVT (pPVT; Penzo et al., 2015). A population of D2 dopamine receptor (*Drd2*)-negative neurons, primarily located in the aPVT, project to the infralimbic region of the medial prefrontal cortex and signal arousal (Gao et al., 2020). Conversely, *Drd2*-positive neurons primarily in the pPVT, project to the prelimbic region of the medial prefrontal cortex and the NAc and are responsive to stimulus valence (Beas et al., 2018; Gao et al., 2020). Due to the topographical and circuit organization of the PVT’s role in behavior that is rapidly emerging in the literature, the impact of various stressors on the PVT’s behavioral roles may also vary. Further, the PVT’s ability to integrate prior experiences with the current context set it up to be a hub for stress memories critical for guiding motivated behaviors. Subsequent sections will discuss what is currently known regarding the activation of the aPVT and pPVT by acute and chronic stressors and their involvement in guiding appropriate behavioral responses. Also, females generally exhibit increased HPA reactivity to an acute stressor compared to males (Weinstock et al., 1998; Bangasser and Valentino, 2014). Given the many known sex differences in the effects of acute and chronic stressors on the brain and behavior but the relative dearth of research examining the female PVT, we will also consider that there may be critical sex differences in the PVT’s role in motivated behavior and effects of stress on PVT physiology and function.

### The Impact of Acute Stress on PVT Activation

The PVT is robustly activated upon exposure to acute stressors and salient stimuli (Zhu et al., 2018; Gao et al., 2020), and PVT activity is sensitive to stress exposure across different stress modalities. Exposure to acute stressors induces increased mRNA expression of the immediate early gene *Fos* or its protein product c-Fos in the PVT of rats, including loud noise (Burow et al., 2005), ether (Emmert and Herman, 1999), cold (Baffi and Palkovits, 2000), open field (Emmert and Herman, 1999), foot shock (Bubser and Deutch, 1999; Brown and Shepard, 2013), forced swim (Cullinan et al., 1995), social defeat (Lkhagvasuren et al., 2014), and physical restraint (Chastrette et al., 1991). Further, distinct stressors can elicit common increases in PVT c-Fos expression, suggesting that the PVT may serve a role of responding to and integrating salient stressors regardless of the specific modality. For example, Baisley et al. (2011) found that both predator (ferret) odor and foot-shock induced similar levels of PVT c-Fos expression. Other physiological stressors like systemically administered drugs of abuse, including cocaine and amphetamine, elicit robust, dose-dependent c-Fos expression in the PVT (Deutch et al., 1998). Food deprivation also increases *Fos* gene expression in both the aPVT and pPVT in obese but not lean Zucker rats (Timofeeva and Richard, 2001), suggesting that the PVT is sensitive to the level of perceived or real stress based on an animal’s prior experience. Collectively, these data suggest that the PVT is poised to respond to many salient stimuli upon first exposure regardless of their valence, and they provide converging evidence for the PVT’s role in motivated behavior across valence domains.

It is important to note that the majority of the work examining acute stress and the PVT has used either male Wistar (Lkhagvasuren et al., 2014; Careaga et al., 2019) or Sprague–Dawley rats (Chastrette et al., 1991; Bubser and Deutch, 1999; Emmert and Herman, 1999; Burow et al., 2005; Brown and Shepard, 2013). However, recent studies show that the PVT also is impacted similarly by acute stress in mice (Beas et al., 2018). While acute stress activation of the PVT has been less well-examined in mice, exposure to a single prolonged stress paradigm (a model of PTSD), consisting of exposures to restraint, swim, predator bedding, and diethyl ether in a single session, increased c-Fos expression in the PVT in C57BL/6 male mice (Azevedo et al., 2020). Differences in strain also have the potential to impact the effect of stressors on the PVT. One study found that acute restraint stress elicited higher PVT c-Fos in BALB/c mice, a more stress-sensitive strain, than C57BL/6 mice (O’Mahony et al., 2010).

And, mouse studies have shown that distinct populations of neurons across the anterior-posterior axis of the PVT may be differentially activated by stressful stimuli. Foot shock stress activates a population of pPVT neurons that project to the NAc (Beas et al., 2018). Multiple aversive stimuli, including foot shock and tail suspension, increase activity in a population of *Drd2*-positive neurons densely expressed in the pPVT, as measured by fiber photometry monitoring of calcium activity. In contrast, these same *Drd2* neurons are inhibited by positively-valenced stimuli such as social interaction and exposure to a thermoneutral zone, suggesting they are specifically activated by negatively-valenced stressful stimuli. *Drd2*-negative neurons that are primarily located in the aPVT, on the other hand, are inhibited by both positively and negatively-valenced stimuli (Gao et al., 2020), indicating their activity is suppressed by salient stimuli regardless of valence. These data are intriguing, as they suggest differential functions and valence sensitivity of the aPVT and pPVT. Data from male rats, however, have shown that exposure to various acute stressors (loud noise, restraint, or foot shock) increases *Fos* mRNA or c-Fos protein expression in both the anterior (Burow et al., 2005) and posterior (Brown and Shepard, 2013; Radley and Sawchenko, 2015) regions of the PVT when specifically examined. Whether differences across studies are due to organizational differences in function between species and strains, methods for monitoring activity, cell-type specificity, and/or other factors is currently unknown.

### Sex Differences in Acute Stress Activation of the PVT

Few studies have examined the impact of acute stress exposure on the PVT and behavior in females, and more work is needed to better understand potential sex differences in the PVT in the acute response to stress. Similarly to males, acute stress exposure increases c-Fos expression in the PVT of females in response to both shaker stress (C57BL/6 mice; Mantella et al., 2004) and immobilization stress (Wistar rats; Ueyama et al., 2006), though direct comparison of c-Fos levels in the PVT in males and females across different stressors has not been performed. Following exposure to an acute stressor, an elevated HPA corticosterone response in females compared to males (Weinstock et al., 1998; Bangasser and Valentino, 2014) could be associated with increased activation of the PVT and differences in PVT-modulated behaviors, though this is not yet established in the existing literature. Following multiple stressor exposures, differences in male and female PVT activation could cause differential plasticity within the PVT and have implications for subsequent behavioral or physiological regulatory activities of the PVT, discussed in “Chronic Stress Effects on the PVT, HPA Axis, and Behavior” section.

Ovarian hormones may further impact acute stress activation of the PVT in females and may differ across the anterior-posterior axis of the PVT, which may be important given that the aPVT and pPVT have been shown to sometimes regulate different motivated behaviors or different aspects of the same motivated behaviors (Ueyama et al., 2006; Do-Monte et al., 2017; Beas et al., 2018; Gao et al., 2020). In a study comparing stress-induced activation of the PVT between ovariectomized (OVX) rats with and without chronic estrogen (estradiol, E2) pellet replacement, acute immobilization stress increased c-Fos expression in the aPVT of OVX but not OVX + E2 rats; in contrast, it increased c-Fos expression in the mid-PVT in OVX + E2 but not OVX rats. Notably, c-Fos expression was unchanged in the pPVT in both groups (Ueyama et al., 2006), contrary to a previously observed immobilization stress-induced c-Fos activation of the pPVT in male rats (Chastrette et al., 1991). The impact of acute stress across the anterior-posterior axis in the PVT is unclear in intact cycling females because this study did not include a sham OVX control group, but these data highlight the ability of E2 to influence stress-induced activation of the PVT in an anatomically distinct manner (Ueyama et al., 2006).

As there are so few studies that include females, it is difficult to compare how the impact of stress on the PVT may differ from males. Drawing conclusions from the few studies including females is especially complicated as species, strain, and type of stressor may differentially impact the acute stress response in males and females. While males may universally exhibit increased PVT activation in response to acute stressor exposure, females may be prone to exhibit differential responses depending on the type of stressor or across the anterior-posterior axis of the PVT. Furthermore, there is evidence from Ueyama et al. (2006) that estrogen has a modulating role on the PVT response to stress (Ueyama et al., 2006), a topic that should be the focus of more extensive future study. Different PVT responses to acute stress in males and females can have implications for plasticity in the PVT following exposure to chronic stressors and impact subsequent stress responsivity in both motivated behaviors and HPA physiology.

## Chronic Stress Effects on The Pvt, Hpa Axis, and Behavior

### Chronic Stress and PVT Regulation of Motivated Behavior

While some motivated behaviors are regulated by the PVT in animals that were not previously exposed to chronic stress (“Acute Stress and PVT Regulation of Motivated Behavior” section), and acute stressors activate the PVT (“The Impact of Acute Stress on PVT Activation” and “Sex Differences in Acute Stress Activation of the PVT” sections), other behavioral roles of the PVT become engaged only following exposure to chronic stressors or repeated experiences (Bhatnagar et al., 2003; Penzo et al., 2015; Zhu et al., 2016; Keyes et al., 2020). The role of the PVT in using past experiences to guide appropriate behavioral responses is illustrated by a study showing that inactivation of the anterior and posterior PVT disrupts appropriate behavior during situations of motivational conflict following a conditioning paradigm in which a conditioned stimulus was paired with first an aversive stimulus and then paired with reward (or vice versa; Choi et al., 2019).

Because the PVT is activated by stressors across modalities and valence (“The Impact of Acute Stress on PVT Activation” section), it may be altered by chronic stress exposure across stress modalities, with implications for regulating different motivated behaviors. For example, creating an association between morphine reward and context over repeated training days in a conditioned place preference paradigm requires the PVT projection to the CeA, while the maintenance of this drug reward association, aversion during withdrawal, and morphine-primed relapse following extinction are dependent on the PVT-NAc pathway (Zhu et al., 2016; Keyes et al., 2020). These data demonstrate the role of the PVT-NAc circuit in drug-related behaviors, particularly following the formation of a drug memory (but see also “Acute Stress and PVT Regulation of Motivated Behavior” section for discussion regarding the PVT-NAc pathway participation in the acquisition of cocaine-seeking behavior). Negatively-valenced learning and memory also involve the PVT in both forming fear memory and in the expression of a fear response. Inactivation of PVT neurons that project to the CeA during either fear conditioning (Penzo et al., 2015) or retrieval (Do-Monte et al., 2015; Penzo et al., 2015) reduces freezing during a retrieval test, and activation of this pathway increases expression of conditioned fear without altering avoidance behavior in novel anxiogenic contexts (Chen and Bi, 2019). These findings suggest that repeated exposure to a stressful or aversive stimulus, such as shock, may be important for the recruitment of the PVT-CeA projection in controlling stable behavioral responses to the stimulus.

These roles for PVT outputs in motivated behavior are likely related to their anatomical location within the PVT. Several studies have shown that the pPVT may be particularly involved in controlling responses to conditioned fear. In male Sprague–Dawley rats trained in a cued fear conditioning paradigm to expect foot shock following the presentation of a tone, pPVT lesions reduce fear expression-freezing following tone presentation (Li et al., 2014). Activating dopamine β-hydroxylase (*Dbh*)-positive locus coeruleus terminals in the pPVT before fear conditioning increases freezing in a retrieval test 24 h later (Beas et al., 2018), suggesting that biogenic amines provide important salience information to the pPVT to promote the consolidation of conditioned fear.

The evidence from the fear literature shows that some chronic stressors can change the way the PVT is engaged by an acute stressor and modulate behavioral responses to it. However, the specific role of the PVT is sensitive to chronic/repeated stress modality, and this too may be specific for varying PVT circuits/subpopulations. In some cases, chronic stress may recruit the PVT to become a modulatory brake on stress reactivity or anxiety-like behavior expression, though the available evidence is limited. For example, pPVT lesions cause increased anxiety-like defensive burying time and height in rats previously exposed to chronic restraint stress compared to non-stressed rats, while either the lesion or chronic stress alone did not impact these behaviors (Bhatnagar et al., 2003). Thus, in many instances, the modulatory role of the PVT in motivated behavior emerges only following repeated exposure to stress-related behavioral stimuli in which integration of these prior experiences is key to appropriate behavioral expression. This recruited engagement of the PVT in behavioral regulation of the stress response could be driven by stress-induced plasticity in the activation or function of the PVT *via* direct HPA-dependent or independent mechanisms, and it may result in an altered role of the PVT in regulating the HPA axis. Whether these lasting effects of chronic stress exposure are HPA-axis dependent or independent is not clear, but bidirectional interaction between the PVT and HPA axis has the potential to impact both behavior and physiology.

### The Impact of Chronic Stress on PVT and HPA Axis Activation

Chronic stress exposure modifies the HPA axis response to an acute stressor, typically measured by blood concentrations of pituitary and adrenal stress hormones. The manner of the alteration depends on the consistency of modality across the chronic and acute stressors. The HPA axis response is typically habituated to an acute stressor that is homotypic to (the same modality as) the chronic paradigm but facilitated when it is heterotypic (novel or a different modality than the chronic stressor), detailed in Table 1 (Bhatnagar and Dallman, 1998; Bhatnagar et al., 2002; Gray et al., 2014; Radley and Sawchenko, 2015). For example, rats exposed to chronic cold stress (Bhatnagar and Dallman, 1998) or chronic variable stress (Radley and Sawchenko, 2015) followed by acute heterotypic restraint stress, exhibit pronounced ACTH and corticosterone responses. Conversely, rats exposed to chronic restraint exhibit blunted ACTH and corticosterone responses to acute homotypic restraint stress (Gray et al., 2014). It is notable, however, that these effects of chronic stress on HPA adaptations are not universal and may be influenced by the type, severity, and pattern of stressors, as well as the specific neuroendocrine endpoint, being assessed (ACTH or CORT; Grissom and Bhatnagar, 2009; Belda et al., 2015).

**Table 1:** The impact of heterotypic and homotypic stress paradigms on the PVT and HPA axis.

Sex	Species/Strain	Age/Weight	Chronic Stressor	Acute Stressor	PVT response vs. no chronic stress	PVT response vs. no acute stress/baseline	HPA response vs. no chronic stress (post-stress timing)	HPA response vs. no acute stress/baseline (post-stress timing)	Citation
Heterotypic stress									
Male	RatSprague-Dawley	200–225g	Cold	Restraint	aPVT: – c-Fos mPVT: – c-Fos pPVT: ↑ c-Fos (60 min)	-	↑ ACTH (15 min) ↑ CORT (30 min)	-	Bhatnagar and Dallman, 1998
Male	Rat Sprague-Dawley albino	2745–325g	Chronic variable stress	Restraint	total: ↓ c-Fos aPVT: ↓ c-Fos pPVT: – c-Fos	↑ ACTH (30 min) ↑ CORT (30, 60, 90 min)	↑ ACTH (30, 60 min) ↑ CORT (30, 60, 90 min)	Radley and Sawchenko, 2015
Male	Rat Sprague-Dawley	190–205g	Defeat	Restraint	-	-	↑ACTH (15 min) ↑CORT (30 min)	-	Bhatnagar and Vining, 2003
Female	Rat Sprague-Dawley	PND 69–71	Defeat	Restraint	PVT: ↓ c-Fos	-	↓ ACTH (baseline)	-	Ver Hoeve et al., 2013
Male	Mouse C57BL/6N	Adult	CORT (4 weeks, drinking water)	Forced Swim	PVT: ↑ c-Fos	-	-	-	Kinlein et al., 2019
Homotypic/Chronic Stress									
Male	Rat Sprague-Dawley	200–225g	Restraint	Restraint	-	-	↓ ACTH (15 min) ↓ CORT (15, 30 min)	-	Bhatnagar et al., 2002
Male	RatSprague-Dawley albino	275–325g	Restraint	Restraint	total: ↓ c-Fos aPVT: ↓ c-Fos pPVT: – c-Fos	total: ↑ c-Fos aPVT: ↑ c-Fos pPVT: ↑ c-Fos	– ACTH – CORT	– ACTH ↑ CORT (30, 60 min)	Radley and Sawchenko, 2015
Male	Rat Sprague-Dawley	54–55 days	Restraint	Restraint	pPVT: ↓ c-Fos vs. acute stress	↓ ACTH (AUC, 0–90 min) ↓ CORT (AUC, 0–90 min)	-	Gray et al., 2014
Male	Rat Sprague-Dawley	70 days	Restraint	Restraint	-	pPVT: – c-Fos	-	-	Lui et al. 2012
Male	Rat Sprague-Dawley	85 days	Restraint	Restraint	-	-	↓ CORT (30, 60 min) ↓ CORT (AUC/min, 0–60 min)	-	Bhatnagar et al., 2005
Female	Rat Sprague-Dawley	105 days	Restraint	Restraint	-	-	↑ CORT (60 min) – CORT (AUC/min, 0–60 min)	-	Bhatnagar et al., 2005
Male	Rat Sprague-Dawley	220–250g	Restraint + GR and MR antagonists in the PVT	Restraint	-	-	↑ ACTH (AUC 0–60 min, vs. vehicle + chronic stress)	-	Jaferi and Bhatnagar, 2006
Male	Rat Sprague-Dawley	200–250g	Intermittent hypoxia	Hypoxia	PVT: ↑ c-Fos	-	-	-	Sica et al. 2000
Male	Rat Wistar	2.5 months	Continuous Social Isolation	-	pPVT: ↑ c-Fos	-	-	-	Stanisavljevic et al., 2019
Male	Rat Sprague-Dawley	6–7 weeks 230–270g	Continuous Flooded Cage	-	aPVT: ↑ c-Fos	-	-	-	Akazawa et al., 2010

Intriguingly, there is evidence that the PVT plays a vital role in this altered HPA axis response, and studies measuring PVT activation and function during acute stress exposure after a history of chronic stress or in rodents with programmed behavioral trait backgrounds provides insight into the relationship between stress, the PVT, and the HPA axis. As discussed in “Acute Stress Effects on the PVT, HPA Axis, and Behavior” section, acute stress activates the PVT, and even after multiple exposures to the same stressor, c-Fos is typically still induced in the PVT (Radley and Sawchenko, 2015). This occurs after chronic restraint (Radley and Sawchenko, 2015) and chronic intermittent hypoxia (Sica et al., 2000; Table 1). There is additional evidence for the roles of the PVT in chronic stress responses from studies that do not specifically measure responses to an acute stressor. Chronic social isolation increases PVT c-Fos (Stanisavljevic et al., 2019), and exposure to a flooded cage increases aPVT activation after chronic exposure, an effect that is reversed after a period of normal housing (Akazawa et al., 2010), suggesting these changes in PVT activation may recover with sufficient time without stress exposure.

However, the level of PVT activation elicited by an acute stressor is often inversely related to the displayed stress reactivity. Male rats bred for high trait anxiety exhibit lower c-Fos expression in the PVT in response to acute exposure to a novel open field compared to rats bred to exhibit low anxiety (Salome et al., 2004). Similarly, following fear conditioning, male rats that exhibit less freezing after context re-exposure 15 days later have higher PVT c-Fos than high-freezers and controls that did not go through training after the second re-exposure to the fear conditioning context (Careaga et al., 2019). Together, these studies suggest that stress-evoked activation of the PVT may suppress the behavioral response to the stressor, discussed in “Chronic Stress and PVT Regulation of Motivated Behavior” section, possibly *via* modulation of the HPA axis.

### PVT Influence on the Neuroendocrine Response to Stress

The inverse relationship between PVT activation and stress reactivity hints at a role for the PVT in suppressing HPA axis activation in response to acute stressors, and interestingly, this modulatory role of the PVT emerges only following chronic stress. Lesion studies show that the PVT has inhibitory activity on the HPA axis whether the acute stressor is homotypic or heterotypic to the chronic stress paradigm. Table 2 details the impact of lesion studies across heterotypic and homotypic stress paradigms. For example, early work characterizing the impact of chronic stress on PVT activation by a heterotypic acute stressor in male rats showed that acute restraint stress elicited a greater HPA axis response in rats with a history of chronic cold stress exposure compared to those without a stress history (Bhatnagar and Dallman, 1998); a follow-up experiment showed that lesioning the pPVT further increased the HPA axis ACTH response to the heterotypic stressor in rats exposed to chronic stress but not in stress-naïve controls (Bhatnagar and Dallman, 1998). These data suggest that acute stressor-induced activation of the PVT serves to suppress the acute HPA axis response only after a history of chronic stress, such that removing this PVT brake disinhibits the HPA axis, leading to an even more robust stress response than that facilitated by heterotypic stress exposure.

**Table 2:** The impact of PVT lesions on the HPA axis and behavior in chronic stress paradigms.

Sex	Species/Strain	Age/Weight	Chronic Stressor	Acute Stressor	Lesion	HPA axis response (post-stress timing, comparison group)	Behavior change (comparison group)	Citation
Male	Sprague-Dawley rat	225–300g	Restraint	Restraint	aPVT	Partial attenuation of CORT habituation (30 min vs. acute only)	-	Fernandes et al. 2002
Male	Sprague-Dawley rat	220–225g	Restraint	Restraint	pPVT	Attenuation of ACTH (15 min) and CORT (15, 30 min) habituation (Day 8 vs. Day 1)	-	Bhatnagar et al., 2002
Male	Sprague-Dawley rat	220–220g	Restraint	Restraint	pPVT	– CORT (30 min, dexamethasone vs. vehicle)	-	Jaferi et al., 2003
Male	Sprague-Dawley rat	200–225g	Cold	Restraint	pPVT	↑ ACTH (30 min vs. sham lesion)	-	Bhatnagar and Dallman, 1998
Male	Sprague-Dawley rat	200–225g	Restraint	-	pPVT	-	↑ defensive burying height and duration (vs. no stress + lesion)	Bhatnagar et al., 2003

The PVT serves the same inhibitory function on HPA axis activation during homotypic chronic stress paradigms, evidenced by one study showing that lesioning the pPVT attenuates HPA axis habituation in a homotypic restraint stress paradigm (Bhatnagar et al., 2002). Together, these lesion studies show that the pPVT has an inhibitory influence on the HPA response to both heterotypic (Bhatnagar and Dallman, 1998) and homotypic (Bhatnagar et al., 2002) stressors, regardless of the chronic stress paradigm, but only in rats with previous exposure to chronic stress. Other groups also find support for the involvement of the aPVT in habituation to homotypic stress. One group showed a partial attenuation of habituation to homotypic restraint stress with an aPVT lesion (Fernandes et al., 2002). These studies suggest that the PVT is recruited after chronic stress to suppress HPA axis activation and potentially influence motivated behavior.

### PVT Activation by Heterotypic and Homotypic Stressors

The evidence from lesion studies showing a role of the PVT in mediating the HPA axis effects of chronic stress is supported by studies assessing c-Fos activation in the PVT in response to homotypic and heterotypic acute stressors following chronic stress, detailed in Table 1. Whether an acute stressor is homotypic or heterotypic to the chronic stress paradigm, PVT c-Fos is usually induced by the acute stressor, but the level of this activation can differ from that evoked in no-chronic stress controls and may depend on subregion within the PVT (Bhatnagar and Dallman, 1998; Radley and Sawchenko, 2015). Radley and Sawchenko (2015) showed that in a heterotypic stress paradigm, the total PVT, driven by the aPVT, displayed decreased c-Fos expression in response to an acute restraint stressor in rats with a history of chronic variable stress compared to rats without. Others have reported that c-Fos expression in the pPVT (but not aPVT) was instead increased in chronically stressed rats following an acute heterotypic stressor (Bhatnagar and Dallman, 1998). However, the same study showed that a pPVT lesion exacerbated the HPA axis facilitation, so increased recruitment, in this case, may reflect a homeostatic upregulation of pPVT control of the HPA axis serving to buffer hyperexcitation.

Overall, the limited evidence available reinforces the implication that PVT activation suppresses acute stress responsivity. This simplistic interpretation suggests that in the case of homotypic stress, PVT activation would be higher in response to an acute stressor in subjects with a history of chronic stress compared to those without. However, many studies find that acute stress activation of the PVT is reduced compared to those without chronic stress in homotypic stress paradigms as it is in heterotypic paradigms. For example, in the same study, Radley and Sawchenko (2015) observed a similar reduction in c-Fos expression in the aPVT and total PVT in response to an acute homotypic restraint stressor following chronic restraint as they did in their similar, but heterotypic, paradigm discussed above (Radley and Sawchenko, 2015). Other groups report a similar decrease in c-Fos expression but in the pPVT (Gray et al., 2014), or they report no increase from baseline at all (Lui et al., 2012), while Radley and Sawchenko (2015), find the pPVT c-Fos expression no different from no-chronic stress control rats in the homotypic stress paradigm (Radley and Sawchenko, 2015).

Specific findings shed light on the intricacies of the relationship between the PVT and HPA axis that are defined by functionally distinct segments of the PVT. Early studies indicated that the pPVT but not the aPVT may be primarily involved in mediating the effects of chronic stress (Bhatnagar and Dallman, 1998; Bhatnagar et al., 2002), and many have focused on the posterior region of the PVT (Bhatnagar and Dallman, 1998; Bhatnagar et al., 2000, 2002, 2003; Bhatnagar and Vining, 2003; Jaferi et al., 2003; Jaferi and Bhatnagar, 2006). Lesion studies have shown that pPVT lesions increase HPA output in chronically stressed rats in both homotypic and heterotypic stress paradigms, however, these studies did not examine the effects of aPVT lesions because their initial experiments uncovered no effects of chronic stress on c-Fos expression in the aPVT (Bhatnagar and Dallman, 1998; Bhatnagar et al., 2002). And as discussed above, Radley and Sawchenko (2015) found that the aPVT (but not pPVT) showed reduced c-Fos activation in response to an acute stressor in both homotypic and heterotypic stress paradigms compared to chronic stress-naïve controls. Together, these studies suggest that the PVT, across its anterior-posterior axis, can be impacted by chronic exposure to stress, but that anatomically distinct populations may be impacted differently by chronic stress and in turn, alter HPA responsivity or motivated behavior differently through distinct PVT circuitry.

While the reduced c-Fos expression in the PVT in homotypic stress paradigms is seemingly in conflict with a hypothesis that PVT activation is inversely related to HPA axis activation in response to acute stress exposure, it reinforces the evidence from the literature suggesting that the PVT is responsive to acute stressors across modalities. And as such, its roles in motivated behaviors may be sensitive to stressors across modalities and timeframes. Further, the variability in findings from these studies speaks to the complexity of the PVT’s organization and function, and they suggest that targeted analysis of specific subpopulations (including those defined by topographical location, molecular class, or circuit organization) may provide insight clarifying the results of studies measuring overall c-Fos activation patterns in the PVT.

### Circuit-Specific Tuning of the PVT

One particularly interesting contrast is that while both the HPA axis and PVT have altered responses to and roles in acute stress responsivity following exposure to chronic stress, the HPA axis response depends on whether the acute stressor is the same (homotypic, habituation) or a different modality (heterotypic, facilitation) than the chronic stressor, while the PVT’s role does not (always inhibitory). These studies suggest that the PVT alone does not control the HPA axis response but that chronic stress engages it to somehow interact with the broader stress control circuity in the brain at the level of the paraventricular nucleus of the thalamus, where the activation of corticotropin-releasing factor (CRF) neurons initiates the HPA axis. However, the PVT has been described to be a hub of stress memory with the ability to directly integrate information about past and current stressors and contexts and control adaptive behavior (Bhatnagar and Dallman, 1998; Hsu et al., 2014). A recent study shows that the PVT is particularly important for appropriate action selection when there is a motivational conflict between cues that previously predicted appetitive (sucrose availability) and aversive (foot shock) stimuli (Choi et al., 2019). This suggests that the PVT is not a simple brake on HPA axis activation but that its role in guiding motivated behavior and stress responses is highly tuned to the past and present circumstances and the type of stimuli and stressors presented. Thus, while the general PVT neuron population may show similar responses to both homotypic and heterotypic stress, specific subpopulations of neurons within the PVT might be sensitive to differences in these stressors.

One potential mechanism through which the PVT may influence HPA axis activity differentially in homotypic and heterotypic stress paradigms is through its glutamatergic (vGlut2-positive) projection to the BNST (Myers et al., 2014), a key limbic target of the PVT primarily consisting of GABAergic projection and interneurons. BNST GABA neurons project to the parvocellular region of the paraventricular nucleus of the hypothalamus (PVN) and can directly inhibit CRF neurons (Dong et al., 2001; Bienkowski and Rinaman, 2011; Crestani et al., 2013; Colmers and Bains, 2018; Song et al., 2020). Therefore, the PVT may be able to provide indirect inhibitory control on the HPA axis *via* the BNST. One study found that in response to an acute novel restraint stressor, rats previously exposed to chronic variable stress (CVS) exhibited lower c-Fos activation in BNST-projecting PVT (PVT-BNST) neurons and PVN-projecting BNST (BNST-PVN) neurons, as well as a potentiated HPA axis response (ACTH and corticosterone), compared to chronic stress-naïve rats (Radley and Sawchenko, 2015). These results suggest that decreased glutamatergic drive from the PVT onto BNST-PVN GABAergic neurons may provide a circuit mechanism for disinhibition of the HPA axis response to heterotypic stress, leading to facilitation of acute stress responsivity. Further, this study found that PVT-BNST neurons did not have decreased c-Fos expression in response to an acute restraint stressor in a homotypic paradigm (even though the total PVT did show decreased c-Fos expression in both paradigms); as such, the intact PVT-BNST-PVN inhibitory brake on the HPA axis may be sufficient to suppress the physiological response to the acute stressor. The differential response in BNST-projectors to heterotypic and homotypic stressors may be one mechanism through which the PVT discriminates between heterotypic and homotypic stress in tuning HPA axis activity.

This study suggests that specific PVT neuron activity becomes an important inhibitor of the HPA axis response to acute stress following chronic stress (Radley and Sawchenko, 2015). Further, this study implicates the BNST, known to inhibit paraventricular hypothalamus CRF neurons that initiate the HPA axis stress response *via* GABAergic projections, as a key target of the PVT neurons modulated by chronic stress. As such, chronic stress may not only recruit the PVT to modulate motivated behavior but also to indirectly inhibit acute stress responsivity *via* the BNST. During homotypic stress, the continued activation of the BNST projection population, as part of a broader network modulating HPA axis function, may be sufficient to support HPA axis habituation. In contrast, reduced activation during heterotypic acute stress may permit HPA axis sensitization through disinhibition *via* the BNST intermediate GABAergic synapse. Altogether, these findings implicate the PVT as a site of chronic stress-induced plasticity across stress exposures that ultimately recruits the PVT to become a modulatory brake on stress responsivity that is sensitive to stress modality, with implications for its role in motivated behavior. However, further characterization of the role of the PVT in modulating motivated behaviors through HPA axis regulation, as well as the potential reciprocal relationship between the effects of chronic stress on the PVT and HPA axis, is necessary.

## Hormone and Neuropeptide Modulation of PVT Function

Overall, following chronic stress, the PVT is activated similarly by an acute stressor regardless of whether it is the same as previously experienced (homotypic) or novel (heterotypic) stressors. The evidence that the PVT is responsive to stressors across modalities, both acutely and following chronic stress, suggests that stress hormones released in response to HPA axis activation broadly across modalities act within the PVT to shape its function. However, the specific subpopulations of PVT neurons, such as those that project to the BNST, are somehow able to tune their responses to homotypic vs. heterotypic stressors after chronic stress, perhaps a learned function that allows for discrimination between and integration of past and new experiences, different threat levels, and other features to guide adaptive behavioral output. The ability of the PVT to respond to yet discriminate between stressors across time may be achieved by either differential stress hormone responses or recruitment of different endogenous hormone and neuropeptide signaling systems across various stressors and chronological presentations. Here we discuss the literature regarding the effects of stress hormones and neuropeptides on the function of the PVT.

## Stress Hormones

Stress hormones including glucocorticoids such as corticosterone/cortisol (rodents/primates, CORT) and mineralocorticoids such as aldosterone may participate in the HPA axis habituation to chronic stress *via* their effects on the PVT. One study found that 4 weeks of chronic CORT administration through drinking water blunts activation of the PVT in response to a subsequent acute stressor (Kinlein et al., 2019), similar to what occurs during habituation to a homotypic stressor (Gray et al., 2014; Radley and Sawchenko, 2015). In line with this, stress-induced adaptations in the PVT that modulate future HPA axis activity are mediated through the glucocorticoid receptors (GR) and mineralocorticoid receptors (MR) in the PVT. Inhibition of GR and MR in the pPVT before each exposure to a chronic stressor prevents HPA axis ACTH habituation to a subsequent acute homotypic restraint stress (Jaferi and Bhatnagar, 2006), suggesting that stress hormone signaling directly in the pPVT is necessary for appropriate HPA axis habituation.

Interestingly, chronic homotypic stress may also elicit a role of the PVT in tuning HPA axis negative feedback. Following chronic homotypic stress, lesioning the pPVT disrupts the ability of the synthetic glucocorticoid dexamethasone to mimic CORT when exogenously administered and provide appropriate negative feedback onto the HPA axis 30 min after the start of acute restraint stress (Jaferi et al., 2003), while this lesion does not impact normal dexamethasone suppression in rats without a history of chronic stress. This may reflect a recruited role of the PVT in the suppression of the HPA axis but only following chronic stress exposure, in line with the inhibitory role of the PVT only following chronic stress observed in earlier lesion studies (Bhatnagar and Dallman, 1998; Bhatnagar et al., 2002).

Together, these data suggest that repeated activation of the HPA axis may lead to chronic stress hormone-induced plasticity in the PVT, causing the structure to be selectively recruited after chronic stress exposure to play new or altered roles in stress responsivity. Repeated exposure to CORT may be one explanation for why the PVT has a similar c-Fos response to both heterotypic and homotypic acute stressors, as CORT is released in response to stressors across modality and paradigm. However, the impact of this repeated exposure to CORT may differ among different PVT circuits, allowing tuning of the HPA axis and discrimination between stress modalities. Further, these findings are particularly intriguing because they suggest that the recruited ability of the PVT to tune HPA axis activation (and thus stress hormone release) in response to acute stress is a direct product of repeated exposure to those same hormones, implicating a reciprocal relationship between the PVT and HPA axis in stress.

## Neuropeptides

Another mechanism through which the PVT can be impacted by chronic stress exposure is through interactions with endogenous neuropeptides. Neuropeptide Y (NPY), αMSH, serotonin, vasopressin, and CRF are all found in PVT (Freedman and Cassell, 1994; Forcelli et al., 2007), and hypothalamic neuropeptidergic neurons, including those that express NPY, cocaine, and amphetamine-regulated transcript (CART), and orexin, project to the PVT (Lee et al., 2015). The role of the orexin/hypocretin system in the PVT has been the most widely studied and extensively reviewed (Martin-Fardon and Boutrel, 2012; Matzeu et al., 2014), while the involvement of other neuropeptides’ signaling and PVT subpopulations have been understudied to date. The literature suggests that orexin/hypocretin in the PVT is involved in modulating behavioral responses in anxiogenic contexts (Li et al., 2010). Specifically, stimulation of hypothalamic orexin neurons in male rats reduces time spent in a social interaction zone and increases c-Fos expression and orexin 1 receptor internalization in the PVT (Heydendael et al., 2014), suggesting that orexin signaling plays a pro-stress role in guiding motivated behavior *via* its actions the PVT. This is supported by direct evidence that site-directed administration of a dual orexin receptor antagonist to the PVT reduces latency to enter a social interaction zone (Dong et al., 2015), and intra-PVT administration of the endogenous ligands for orexin receptors, orexin A and orexin B, each increase avoidance of the open arms of the elevated plus-maze (Li et al., 2010).

There is also a role for the actions of orexin in the PVT in balancing the behavioral response to natural and drug rewards (Matzeu et al., 2014, 2018). For example, intra-pPVT orexin-A delivery reinstates self-administration of cocaine or sweetened condensed milk following the conditioning and extinction of this behavior (Matzeu et al., 2018). However, simultaneous dynorphin A administration to activate endogenous kappa opioid receptors blocks cocaine but not sweetened condensed milk reinstatement, indicating a complex interaction between orexin and dynorphin signaling in the pPVT in the regulation of drug-related appetitive motivated behavior that is dependent on prior repeated exposures and learned associations between drug rewards and the cues that predict them (Matzeu et al., 2018). On top of its role in motivated behavior, PVT orexin signaling may be a key component of the PVT’s recruited role in modulating acute HPA axis stress responses. For example, administration of an orexin receptor antagonist during each of four daily forced swim stressors in the pPVT attenuates the facilitation of the HPA axis response to acute heterotypic restraint stress on the fifth day (Heydendael et al., 2011).

There is limited evidence about the role of other neuropeptide systems in the PVT, suggesting that there may be many more signaling mechanisms for stress-induced changes in the PVT and its role in motivated behavior and acute stress responsivity. Acute stress increases nociception/orphanin FQ mRNA in the PVT (Zambello et al., 2008), but it is unknown whether this undergoes plasticity with chronic stress or has an impact on behavior. Whether and how other neuropeptides from the hypothalamus and other distal and intra-PVT neurons modulate PVT function acutely and after chronic stress exposure remains to be examined. And, as previously discussed, current studies almost exclusively use male rats. Further study of the impact of these neuropeptidergic systems in the PVT in females is especially important as sex differences have been found in the consequences of chronic stress and exercise on orexin neurons in the hypothalamus (James et al., 2014) and after chronic stress alone on hypocretin (orexin)-1 receptor gene expression in the prefrontal cortex (Lu et al., 2017). There are still many remaining questions about the role of neuropeptides in the PVT on HPA axis activity and motivated behavior, including whether neuropeptides interact with stress hormones in the PVT’s recruited role in heterotypic and homotypic stress responses. But together, these studies suggest that neuropeptides could influence the tuning of HPA axis activity through the PVT. These neuropeptides may be recruited by different stressors depending on the stress modality, severity, or pattern, allowing the PVT to tune its function to discriminate among different stress modalities and behavioral paradigms, potentially impacting different motivated behaviors.

## Sex Differences in Chronic Stress Effects on the PVT and HPA Axis

The majority of studies examining the role of chronic stress on PVT and HPA axis activation occur in male Sprague–Dawley rats (Bhatnagar and Dallman, 1998; Sica et al., 2000; Bhatnagar et al., 2002, 2003; Bhatnagar and Vining, 2003; Lui et al., 2012; Gray et al., 2014). It is therefore unclear how these findings of the effects of homotypic and heterotypic stress paradigms on the PVT and HPA axis can be extended to females, as well as to other species and strains. Here we summarize what is known about the chronic stress impact on the PVT and HPA axis in females and how they compare to findings in males.

### Chronic Stress in Adulthood

Sex differences in the HPA axis response to acute and chronic stress have been well established in the literature and reviewed by others (Goel et al., 2011; Bangasser and Valentino, 2014; Green and McCormick, 2016), but the role of the PVT in the sex-specific consequences of stress is less clear. Male rats exposed to chronic defeat and then heterotypic restraint exhibit facilitation of their HPA response to the acute restraint compared to non-stressed controls (Bhatnagar and Vining, 2003). However, adult female rats exposed to a similar paradigm do not exhibit differences in the CORT response following exposure to an acute restraint stressor compared to those without a history of chronic stress, though they do have blunted ACTH at baseline (Ver Hoeve et al., 2013). These studies suggest that chronic stress has a sex-dependent effect on future HPA activity that may be related to more robust stress-induced adaptations in the PVT of males than females, at least for heterotypic stress. Furthermore, one group shows that exposure to a homotypic restraint paradigm results in more robust habituation of the HPA axis response in males than in females (Bhatnagar et al., 2005), suggesting that females may not as readily adapt to repeated stress exposure; however, sex differences in HPA axis habituation across studies do not always show this exact pattern, as previously reviewed (Heck and Handa, 2019).

Activation of the PVT in chronic stress paradigms may also differ between males and females. The c-Fos expression is increased in the PVT of female mice following exposure to an acute stressor (Mantella et al., 2004), as has been shown in males. However, while male rats with a history of chronic stress exposure subsequently exposed to a heterotypic stressor (chronic cold exposure followed by acute restraint) exhibit increased PVT c-Fos expression compared to controls that were not chronically stressed (Bhatnagar and Dallman, 1998), female rats exposed to a heterotypic stressor (social defeat followed by restraint) do not exhibit different acute stress-induced PVT activation compared to non-stressed control rats (Ver Hoeve et al., 2013). Whether this difference is due to a sex difference or due to the use of a chronic defeat paradigm is unclear, but males in a different study exposed to a chronic variable stress paradigm exhibit reduced c-Fos expression in the anterior and whole PVT following novel stressor exposure compared to stress-naïve controls (Radley and Sawchenko, 2015). These data suggest that plasticity within the PVT following chronic stress exposure may differ in males and females. As the PVT is involved in both regulating motivated behavior and the HPA axis, differences in PVT plasticity could manifest in sex-specific vulnerability to chronic stress-induced disruptions in motivated behavior, perhaps more robustly in males.

### Chronic Stress During Development

Research from developmental stress models suggests that stress affects the activation of the PVT from an early age and that the female PVT may be more sensitive to developmental stress than the male PVT, though the evidence is limited. pPVT activity is observed in newborn rat pups (Gibbs et al., 1990), suggesting a developmental role of pPVT activity that could be disrupted with early life stress exposure. One study examining early life conditions found that female rats raised in large litters with reduced access to food and maternal care compared to those raised in smaller litters exhibited higher anxiety-like behavior in adulthood and reduced PVT activation in response to acute stress, suggesting a heterotypic-like stress effect on the female PVT (Spencer and Tilbrook, 2009). On the other hand, males in this same study did not exhibit differences in acute stress-induced PVT activation (Spencer and Tilbrook, 2009), suggesting that females are more susceptible to early life stress. Another study showed that adolescent male Sprague–Dawley rats exposed to chronic stress followed by a heterotypic or homotypic stressor show similar pPVT c-Fos expression in response to the acute stressors as a cohort of males that underwent the same paradigm in adulthood (Lui et al., 2012). These data suggest that the effects of chronic stress on the male PVT and its response to acute stressors are fairly stable across development, while female responses are more sensitive to the developmental stage.

In addition to sex- and age-dependent effects of chronic stress, the reversibility of these effects may also diverge in males and females. When male and female rats were exposed to early life stress, males given a running wheel in late adolescence expressed a more robust PVT c-Fos activation in response to an acute restraint stressor than controls and those exposed to early life stress alone; in contrast, females exhibited a less robust c-Fos response than the other groups, showing that chronic early life stress can cause sex-dependent PVT plasticity (James et al., 2014). These male rats also exhibited a behavioral recovery of early life stress effects when given a running wheel, while females did not, providing a link between differential PVT activity and the impact of stress on behavior and suggesting that the reversal of developmental stress effects on the PVT is more achievable in males than females (James et al., 2014). This inability of PVT plasticity to recover hints at a potential mechanism for females’ increased susceptibility to stress-induced neuropsychiatric disease phenotypes in humans.

Future studies are necessary to further dissect the role of the PVT in the intricate, sex-specific HPA axis-PVT relationship during heterotypic and homotypic stress. Though data about the impact of chronic stress on the activity of PVT in females is limited, these studies suggest that PVT adaptations to chronic stress may differ in males and females, particularly in their response to novel acute stressors, and depend on the developmental stage. Bhatnagar and colleagues propose that the potentiated HPA and PVT response in males exposed to chronic stress and a heterotypic stressor allows an organism to integrate previous information to adequately respond to a novel threat (Bhatnagar and Dallman, 1998; Hsu et al., 2014); it is, therefore, possible that this adaptation is disrupted or is mechanistically different in females and has potential implications on the future stress response. Differential adaptations following chronic stress in the PVT in males and females that alter the activity of the HPA axis can have sex-specific consequences on motivated behaviors through both PVT and HPA axis-driven mechanisms.

## Conclusions

The PVT is an important regulator of motivated behavior, and additional regulatory roles of the PVT emerge only after exposure to repeated behaviors or stimuli; this is particularly pronounced following repeated exposure to stress. PVT activity is both responsive to acute and chronic stress and has the ability, particularly following chronic stress exposure, to regulate the HPA axis response to acute stress. Furthermore, distinct neuronal populations within the PVT have independent roles in guiding future motivated behavior and the neuroendocrine stress response and may be impacted differently by chronic stress. Due to the complexity of the PVT’s organization and its impact on motivated behavior, the potential consequences of stress on the PVT’s role in behavioral control are equally complex. Recent studies using fiber photometry have shown stress-responsive activity of PVT neurons in discrete circuitry, and optogenetic and chemogenetic studies have shown the complexity of the PVT’s role in motivated behavior through modulation of distinct behaviors through projections to different target regions. However, the impact of chronic stress in these distinct circuits and the potential impact on behavior is still understudied. Increased specificity with a future study focusing on the impact of heterotypic and homotypic stress paradigms in distinct PVT circuitry will provide more precise insight into the impact of stress on the PVT’s role in motivated behaviors. Furthermore, an improved understanding of the impact of stress hormones and neuropeptides within these circuits in heterotypic and homotypic stress paradigms is important because signaling mechanisms are involved in aberrant behavioral responses to novel or acute stimuli following exposure to stress are relevant for understanding stress-related neuropsychiatric disease. Future research on the PVT’s role in heterotypic and homotypic stress paradigms will have a greater impact on understanding mechanisms of neuropsychiatric disease than studying these interacting components independently. This may be particularly important in females, who have increased susceptibility to stress-related neuropsychiatric diseases, and a more complete understanding of sex differences in the impact of heterotypic and homotypic stress on the PVT and its role in motivated behaviors is necessary.

## Author Contributions

SAR and KEP wrote and edited the manuscript. All authors contributed to the article and approved the submitted version.

## Conflict of Interest

The authors declare that the research was conducted in the absence of any commercial or financial relationships that could be construed as a potential conflict of interest.

## References

[B1] Akazawa K. H. Cui Y. Tanaka M. Kataoka Y. Yoneda Y. Watanabe Y.. (2010). Mapping of regional brain activation in response to fatigue-load and recovery in rats with c-Fos immunohistochemistry. Neurosci. Res. 66, 372–379. 10.1016/j.neures.2009.12.009 20018215

[B2] Amir A. Paré J. F. Smith Y. Paré D. (2019). Midline thalamic inputs to the amygdala: ultrastructure and synaptic targets. J. Comp. Neurol. 527, 942–956. 10.1002/cne.24557 30311651 PMC6347509

[B3] Azevedo H. Ferreira M. Mascarello A. Osten P. Guimaraes C. R. W. (2020). Brain-wide mapping of c-fos expression in the single prolonged stress model and the effects of pretreatment with ACH-000029 or prazosin. Neurobiol. Stress 13:100226. 10.1016/j.ynstr.2020.100226 32478146 PMC7251424

[B4] Baffi J. S. Palkovits M. (2000). Fine topography of brain areas activated by cold stress. A fos immunohistochemical study in rats. Neuroendocrinology 72, 102–113. 10.1159/000054577 10971145

[B5] Baisley S. K. Cloninger C. L. Bakshi V. P. (2011). Fos expression following regimens of predator stress versus footshock that differentially affect prepulse inhibition in rats. Physiol. Behav. 104, 796–803. 10.1016/j.physbeh.2011.08.001 21843541 PMC3183172

[B6] Bangasser D. A. Valentino R. J. (2014). Sex differences in stress-related psychiatric disorders: neurobiological perspectives. Front. Neuroendocrinol. 35, 303–319. 10.1016/j.yfrne.2014.03.008 24726661 PMC4087049

[B7] Beas B. S. Wright B. J. Skirzewski M. Leng Y. Hyun J. H. Koita O.. (2018). The locus coeruleus drives disinhibition in the midline thalamus *via* a dopaminergic mechanism. Nat. Neurosci. 21, 963–973. 10.1038/s41593-018-0167-4 29915192 PMC6035776

[B8] Belda X. Fuentes S. Daviu N. Nadal R. Armario A. (2015). Stress-induced sensitization: the hypothalamic-pituitary-adrenal axis and beyond. Stress 18, 269–279. 10.3109/10253890.2015.1067678 26300109

[B10] Bhatnagar S. Dallman M. (1998). Neuroanatomical basis for facilitation of hypothalamic-pituitary-adrenal responses to a novel stressor after chronic stress. Neuroscience 84, 1025–1039. 10.1016/s0306-4522(97)00577-0 9578393

[B11] Bhatnagar S. Huber R. Lazar E. Pych L. Vining C. (2003). Chronic stress alters behavior in the conditioned defensive burying test: role of the posterior paraventricular thalamus. Pharmacol. Biochem. Behav. 76, 343–349. 10.1016/j.pbb.2003.08.005 14592687

[B12] Bhatnagar S. Huber R. Nowak N. Trotter P. (2002). Lesions of the posterior paraventricular thalamus block habituation of hypothalamic-pituitary-adrenal responses to repeated restraint. J. Neuroendocrinol. 14, 403–410. 10.1046/j.0007-1331.2002.00792.x 12000546

[B13] Bhatnagar S. Lee T. M. Vining C. (2005). Prenatal stress differentially affects habituation of corticosterone responses to repeated stress in adult male and female rats. Horm. Behav. 47, 430–438. 10.1016/j.yhbeh.2004.11.019 15777808

[B14] Bhatnagar S. Viau V. Chu A. Soriano L. Meijer O. C. Dallman M. F.. (2000). A cholecystokin in-mediated pathway to the paraventricular thalamus is recruited in chronically stressed rats and regulates hypothalamic-pituitary-adrenal function. J. Neurosci. 20, 5564–5573. 10.1523/JNEUROSCI.20-14-05564.2000 10884340 PMC6772345

[B9] Bhatnagar S. Vining C. (2003). Facilitation of hypothalamic-pituitary-adrenal responses to novel stress following repeated social stress using the resident/intruder paradigm. Horm. Behav. 43, 158–165. 10.1016/s0018-506x(02)00011-9 12614646

[B15] Bienkowski M. S. Rinaman L. (2011). Immune challenge activates neural inputs to the ventrolateral bed nucleus of the stria terminalis. Physiol. Behav. 104, 257–265. 10.1016/j.physbeh.2011.03.006 21402087 PMC3118915

[B16] Brown P. L. Shepard P. D. (2013). Lesions of the fasciculus retroflexus alter footshock-induced cFos expression in the mesopontine rostromedial tegmental area of rats. PLoS One 8:e60678. 10.1371/journal.pone.0060678 23593280 PMC3625179

[B17] Bubser M. Deutch A. Y. (1999). Stress induces Fos expression in neurons of the thalamic paraventricular nucleus that innervate limbic forebrain sites. Synapse 32, 13–22. 10.1002/(SICI)1098-2396(199904)32:1<13::AID-SYN2>3.0.CO;2-R 10188633

[B18] Burow A. Day H. E. Campeau S. (2005). A detailed characterization of loud noise stress: intensity analysis of hypothalamo-pituitary-adrenocortical axis and brain activation. Brain Res. 1062, 63–73. 10.1016/j.brainres.2005.09.031 16256084 PMC2409188

[B19] Careaga M. B. L. Girardi C. E. N. Suchecki D. (2019). Variability in response to severe stress: highly reactive rats exhibit changes in fear and anxiety-like behavior related to distinct neuronal co-activation patterns. Behav. Brain Res. 373:112078. 10.1016/j.bbr.2019.112078 31336139

[B20] Chastrette N. Pfaff D. W. Gibbs R. B. (1991). Effects of daytime and nighttime stress on Fos-like immunoreactivity in the paraventricular nucleus of the hypothalamus, the habenula and the posterior paraventricular nucleus of the thalamus. Brain Res. 563, 339–344. 10.1016/0006-8993(91)91559-j 1786549

[B21] Chen M. Bi L. L. (2019). Optogenetic long-term depression induction in the PVT-CeL circuitry mediates decreased fear memory. Mol. Neurobiol. 56, 4855–4865. 10.1007/s12035-018-1407-z 30406427

[B22] Cheng J. Wang J. Ma X. Ullah R. Shen Y. Zhou Y. D. (2018). Anterior paraventricular thalamus to nucleus accumbens projection is involved in feeding behavior in a novel environment. Front. Mol. Neurosci. 11:202. 10.3389/fnmol.2018.00202 29930498 PMC5999750

[B24] Choi E. A. Jean-Richard-Dit-Bressel P. Clifford C. W. G. McNally G. P. (2019). Paraventricular thalamus controls behavior during motivational conflict. J. Neurosci. 39, 4945–4958. 10.1523/JNEUROSCI.2480-18.2019 30979815 PMC6670259

[B23] Choi E. A. McNally G. P. (2017). Paraventricular thalamus balances danger and reward. J. Neurosci. 37, 3018–3029. 10.1523/JNEUROSCI.3320-16.2017 28193686 PMC6596734

[B25] Colmers P. L. W. Bains J. S. (2018). Balancing tonic and phasic inhibition in hypothalamic corticotropin-releasing hormone neurons. J. Physiol. 596, 1919–1929. 10.1113/JP275588 29419884 PMC5978301

[B26] Crestani C. C. Alves F. H. Gomes F. V. Resstel L. B. Correa F. M. Herman J. P. (2013). Mechanisms in the bed nucleus of the stria terminalis involved in control of autonomic and neuroendocrine functions: a review. Curr. Neuropharmacol. 11, 141–159. 10.2174/1570159X11311020002 23997750 PMC3637669

[B27] Cullinan W. E. Herman J. P. Battaglia D. F. Akil H. Watson S. J. (1995). Pattern and time course of immediate early gene expression in rat brain following acute stress. Neuroscience 64, 477–505. 10.1016/0306-4522(94)00355-9 7700534

[B28] Deutch A. Y. Bubser M. Young C. D. (1998). Psychostimulant-induced Fos protein expression in the thalamic paraventricular nucleus. J. Neurosci. 18, 10680–10687. 10.1523/JNEUROSCI.18-24-10680.1998 9852603 PMC6793371

[B29] Do-Monte F. H. Minier-Toribio A. Quiñones-Laracuente K. Medina-Colón E. M. Quirk G. J. (2017). Thalamic regulation of sucrose seeking during unexpected reward omission. Neuron 94, e4388–e4400. 10.1016/j.neuron.2017.03.036 28426970 PMC5484638

[B30] Do-Monte F. H. Quiñones-Laracuente K. Quirk G. J. (2015). A temporal shift in the circuits mediating retrieval of fear memory. Nature 519, 460–463. 10.1038/nature14030 25600268 PMC4376623

[B31] Dong H. W. Petrovich G. D. Watts A. G. Swanson L. W. (2001). Basic organization of projections from the oval and fusiform nuclei of the bed nuclei of the stria terminalis in adult rat brain. J. Comp. Neurol. 436, 430–455. 10.1002/cne.1079 11447588

[B32] Dong X. Li Y. Kirouac G. J. (2015). Blocking of orexin receptors in the paraventricular nucleus of the thalamus has no effect on the expression of conditioned fear in rats. Front. Behav Neurosci. 9:161. 10.3389/fnbeh.2015.00161 26136671 PMC4468823

[B33] Emmert M. H. Herman J. P. (1999). Differential forebrain c-fos mRNA induction by ether inhalation and novelty: evidence for distinctive stress pathways. Brain Res. 845, 60–67. 10.1016/s0006-8993(99)01931-9 10529444

[B34] Fernandes G. A. Perks P. Cox N. K. Lightman S. L. Ingram C. D. Shanks N. (2002). Habituation and cross-sensitization of stress-induced hypothalamic-pituitary-adrenal activity: effect of lesions in the paraventricular nucleus of the thalamus or bed nuclei of the stria terminalis. J. Neuroendocrinol. 14, 593–602. 10.1046/j.1365-2826.2002.00819.x 12121498

[B35] Forcelli P. A. Orefice L. L. Heinrichs S. C. (2007). Neural, endocrine and electroencephalographic hyperreactivity to human contact: a diathesis-stress model of seizure susceptibility in El mice. Brain Res. 1144, 248–256. 10.1016/j.brainres.2007.01.100 17320061

[B36] Freedman L. J. Cassell M. D. (1994). Relationship of thalamic basal forebrain projection neurons to the peptidergic innervation of the midline thalamus. J. Comp. Neurol. 348, 321–342. 10.1002/cne.903480302 7844251

[B37] Gao C. Leng Y. Ma J. Rooke V. Rodriguez-Gonzalez S. Ramakrishnan C.. (2020). Two genetically, anatomically and functionally distinct cell types segregate across anteroposterior axis of paraventricular thalamus. Nat. Neurosci. 23, 217–228. 10.1038/s41593-019-0572-3 31932767 PMC7007348

[B38] Gibbs R. B. Lombardino A. Pfaff D. W. (1990). Sex steroids and fos expression in the CNS of prepubertal and newborn rats. Mol. Cell. Neurosci. 1, 250–261. 10.1016/1044-7431(90)90007-q 19912775

[B39] Goel N. Plyler K. S. Daniels D. Bale T. L. (2011). Androgenic influence on serotonergic activation of the HPA stress axis. Endocrinology 152, 2001–2010. 10.1210/en.2010-0964 21385938 PMC3075941

[B40] Gray M. Innala L. Viau V. (2014). Central vasopressin V1A receptor blockade alters patterns of cellular activation and prevents glucocorticoid habituation to repeated restraint stress exposure. Int. J. Neuropsychopharmacol. 17, 2005–2015. 10.1017/S1461145714000935 24913767

[B41] Green M. R. McCormick C. M. (2016). Sex and stress steroids in adolescence: gonadal regulation of the hypothalamic-pituitary-adrenal axis in the rat. Gen. Comp. Endocrinol. 234, 110–116. 10.1016/j.ygcen.2016.02.004 26851306

[B42] Grissom N. Bhatnagar S. (2009). Habituation to repeated stress: get used to it. Neurobiol. Learn. Mem. 92, 215–224. 10.1016/j.nlm.2008.07.001 18667167 PMC2773683

[B43] Heck A. L. Handa R. J. (2019). Sex differences in the hypothalamic-pituitary-adrenal axis’ response to stress: an important role for gonadal hormones. Neuropsychopharmacology 44, 45–58. 10.1038/s41386-018-0167-9 30111811 PMC6235871

[B44] Heydendael W. Sengupta A. Beck S. Bhatnagar S. (2014). Optogenetic examination identifies a context-specific role for orexins/hypocretins in anxiety-related behavior. Physiol. Behav. 130, 182–190. 10.1016/j.physbeh.2013.10.005 24140988 PMC4155939

[B45] Heydendael W. Sharma K. Iyer V. Luz S. Piel D. Beck S.. (2011). Orexins/hypocretins act in the posterior paraventricular thalamic nucleus during repeated stress to regulate facilitation to novel stress. Endocrinology 152, 4738–4752. 10.1210/en.2011-1652 21971160 PMC3230061

[B46] Hsu D. T. Kirouac G. J. Zubieta J. K. Bhatnagar S. (2014). Contributions of the paraventricular thalamic nucleus in the regulation of stress, motivation and mood. Front. Behav. Neurosci. 8:73. 10.3389/fnbeh.2014.00073 24653686 PMC3949320

[B47] Hua R. Wang X. Chen X. Huang P. Li P. Mei W.. (2018). Calretinin neurons in the midline thalamus modulate starvation-induced arousal. Curr. Biol. 28, e43948–e43959. 10.1016/j.cub.2018.11.020 30528578

[B48] Jaferi A. Bhatnagar S. (2006). Corticosterone can act at the posterior paraventricular thalamus to inhibit hypothalamic-pituitary-adrenal activity in animals that habituate to repeated stress. Endocrinology 147, 4917–4930. 10.1210/en.2005-1393 16809449

[B49] Jaferi A. Nowak N. Bhatnagar S. (2003). Negative feedback functions in chronically stressed rats: role of the posterior paraventricular thalamus. Physiol. Behav. 78, 365–373. 10.1016/s0031-9384(03)00014-3 12676271

[B50] James M. H. Campbell E. J. Walker F. R. Smith D. W. Richardson H. N. Hodgson D. M.. (2014). Exercise reverses the effects of early life stress on orexin cell reactivity in male but not female rats. Front. Behav. Neurosci. 8:244. 10.3389/fnbeh.2014.00244 25100956 PMC4107856

[B51] Keyes P. C. Adams E. L. Chen Z. Bi L. Nachtrab G. Wang V. J.. (2020). Orchestrating opiate-associated memories in thalamic circuits. Neuron 107, 1113–1123. 10.1016/j.neuron.2020.06.028 32679036 PMC8130576

[B52] Kinlein S. A. Phillips D. J. Keller C. R. Karatsoreos I. N. (2019). Role of corticosterone in altered neurobehavioral responses to acute stress in a model of compromised hypothalamic-pituitary-adrenal axis function. Psychoneuroendocrinology 102, 248–255. 10.1016/j.psyneuen.2018.12.010 30594817 PMC7649055

[B53] Kirouac G. J. (2015). Placing the paraventricular nucleus of the thalamus within the brain circuits that control behavior. Neurosci. Biobehav. Rev. 56, 315–329. 10.1016/j.neubiorev.2015.08.005 26255593

[B54] Lee J. S. Lee E. Y. Lee H. S. (2015). Hypothalamic, feeding/arousal-related peptidergic projections to the paraventricular thalamic nucleus in the rat. Brain Res. 1598, 97–113. 10.1016/j.brainres.2014.12.029 25529631

[B55] Levine O. B. Skelly M. J. Miller J. F. DiBerto S. A. Rowson J. K. Rivera-Irizarry T. L.. (2020). The paraventricular thalamus provides a polysynaptic brake on limbic CRF neurons to sex-dependently blunt binge alcohol drinking and avoidance behavior. bioRxiv [Preprint]. 10.1101/2020.05.04.075051 PMC8382748 34426574

[B56] Li Y. Li S. Wei C. Wang H. Sui N. Kirouac G. J. (2010). Orexins in the paraventricular nucleus of the thalamus mediate anxiety-like responses in rats. Psychopharmacology 212, 251–265. 10.1007/s00213-010-1948-y 20645079

[B57] Li Y. Dong X. Li S. Kirouac G. J. (2014). Lesions of the posterior paraventricular nucleus of the thalamus attenuate fear expression. Front. Behav. Neurosci. 8:94. 10.3389/fnbeh.2014.00094 24688461 PMC3960725

[B58] Lkhagvasuren B. Oka T. Nakamura Y. Hayashi H. Sudo N. Nakamura K. (2014). Distribution of Fos-immunoreactive cells in rat forebrain and midbrain following social defeat stress and diazepam treatment. Neuroscience 272, 34–57. 10.1016/j.neuroscience.2014.04.047 24797330

[B59] Lu J. Zhao J. Balesar R. Fronczek R. Zhu Q. B. Wu X. Y.. (2017). Sexually dimorphic changes of hypocretin (Orexin) in depression. EBioMedicine 18, 311–319. 10.1016/j.ebiom.2017.03.043 28377228 PMC5405188

[B60] Lui P. Padow V. A. Franco D. Hall B. S. Park B. Klein Z. A.. (2012). Divergent stress-induced neuroendocrine and behavioral responses prior to puberty. Physiol. Behav. 107, 104–111. 10.1016/j.physbeh.2012.06.011 22728428 PMC3409319

[B61] Mantella R. C. Vollmer R. R. Rinaman L. Li X. Amico J. A. (2004). Enhanced corticosterone concentrations and attenuated Fos expression in the medial amygdala of female oxytocin knockout mice exposed to psychogenic stress. Am. J. Physiol. Regul. Integr. Comp. Physiol. 287, R1494–R1504. 10.1152/ajpregu.00387.2004 15319220

[B62] Martin-Fardon R. Boutrel B. (2012). Orexin/hypocretin (Orx/Hcrt) transmission and drug-seeking behavior: is the paraventricular nucleus of the thalamus (PVT) part of the drug seeking circuitry. Front. Behav. Neurosci. 6:75. 10.3389/fnbeh.2012.00075 23162448 PMC3494007

[B64] Matzeu A. Kallupi M. George O. Schweitzer P. Martin-Fardon R. (2018). Dynorphin counteracts orexin in the paraventricular nucleus of the thalamus: cellular and behavioral evidence. Neuropsychopharmacology 43, 1010–1020. 10.1038/npp.2017.250 29052613 PMC5854806

[B63] Matzeu A. Zamora-Martinez E. R. Martin-Fardon R. (2014). The paraventricular nucleus of the thalamus is recruited by both natural rewards and drugs of abuse: recent evidence of a pivotal role for orexin/hypocretin signaling in this thalamic nucleus in drug-seeking behavior. Front. Behav. Neurosci. 8:117. 10.3389/fnbeh.2014.00117 24765071 PMC3982054

[B65] Millan E. Z. Ong Z. McNally G. P. (2017). Paraventricular thalamus: gateway to feeding, appetitive motivation and drug addiction. Prog. Brain Res. 235, 113–137. 10.1016/bs.pbr.2017.07.006 29054285

[B66] Myers B. Mark Dolgas C. Kasckow J. Cullinan W. E. Herman J. P. (2014). Central stress-integrative circuits: forebrain glutamatergic and GABAergic projections to the dorsomedial hypothalamus, medial preoptic area and bed nucleus of the stria terminalis. Brain Struct. Funct. 219, 1287–1303. 10.1007/s00429-013-0566-y 23661182 PMC3778169

[B67] Neumann P. A. Wang Y. Yan Y. Ishikawa M. Cui R. Huang Y. H.. (2016). Cocaine-induced synaptic alterations in thalamus to nucleus accumbens projection. Neuropsychopharmacology 41, 2399–2410. 10.1038/npp.2016.52 27074816 PMC4946070

[B68] O’Mahony C. M. Sweeney F. F. Daly E. Dinan T. G. Cryan J. F. (2010). Restraint stress-induced brain activation patterns in two strains of mice differing in their anxiety behaviour. Behav. Brain Res. 213, 148–154. 10.1016/j.bbr.2010.04.038 20435071

[B69] Penzo M. A. Robert V. Tucciarone J. De Bundel D. Wang M. Van Aelst L.. (2015). The paraventricular thalamus controls a central amygdala fear circuit. Nature 519, 455–459. 10.1038/nature13978 25600269 PMC4376633

[B70] Radley J. J. Sawchenko P. E. (2015). Evidence for involvement of a limbic paraventricular hypothalamic inhibitory network in hypothalamic-pituitary-adrenal axis adaptations to repeated stress. J. Comp. Neurol. 523, 2769–2787. 10.1002/cne.23815 26010947 PMC4607561

[B71] Salome N. Salchner P. Viltart O. Sequeira H. Wigger A. Landgraf R.. (2004). Neurobiological correlates of high (HAB) versus low anxiety-related behavior (LAB): differential Fos expression in HAB and LAB rats. Biol. Psychiatry 55, 715–723. 10.1016/j.biopsych.2003.10.021 15039000

[B72] Sica A. L. Greenberg H. E. Scharf S. M. Ruggiero D. A. (2000). Chronic-intermittent hypoxia induces immediate early gene expression in the midline thalamus and epithalamus. Brain Res. 883, 224–228. 10.1016/s0006-8993(00)02800-6 11074051

[B73] Song Y. Meng Q. X. Wu K. Hua R. Song Z. J. Qin X.. (2020). Disinhibition of PVN-projecting GABAergic neurons in AV region in BNST participates in visceral hypersensitivity in rats. Psychoneuroendocrinology 117:104690. 10.1016/j.psyneuen.2020.104690 32417623

[B74] Spencer S. J. Tilbrook A. (2009). Neonatal overfeeding alters adult anxiety and stress responsiveness. Psychoneuroendocrinology 34, 1133–1143. 10.1016/j.psyneuen.2009.02.013 19303720 PMC2726293

[B75] Stanisavljevic A. Peric I. Bernardi R. E. Gass P. Filipovic D. (2019). Clozapine increased c-Fos protein expression in several brain subregions of socially isolated rats. Brain Res. Bull. 152, 35–44. 10.1016/j.brainresbull.2019.07.005 31299320

[B76] Timofeeva E. Richard D. (2001). Activation of the central nervous system in obese Zucker rats during food deprivation. J. Comp. Neurol. 441, 71–89. 10.1002/cne.1398 11745636

[B77] Ueyama T. Tanioku T. Nuta J. Kujira K. Ito T. Nakai S.. (2006). Estrogen alters c-Fos response to immobilization stress in the brain of ovariectomized rats. Brain Res. 1084, 67–79. 10.1016/j.brainres.2006.02.008 16545785

[B78] Ver Hoeve E. S. Kelly G. Luz S. Ghanshani S. Bhatnagar S. (2013). Short-term and long-term effects of repeated social defeat during adolescence or adulthood in female rats. Neuroscience 249, 63–73. 10.1016/j.neuroscience.2013.01.073 23402852 PMC3743933

[B79] Weinstock M. Razin M. Schorer-Apelbaum D. Men D. McCarty R. (1998). Gender differences in sympathoadrenal activity in rats at rest and in response to footshock stress. Int. J. Dev. Neurosci. 16, 289–295. 10.1016/s0736-5748(98)00021-5 9785125

[B80] Zambello E. Jimenez-Vasquez P. A. El Khoury A. Mathe A. A. Caberlotto L. (2008). Acute stress differentially affects corticotropin-releasing hormone mRNA expression in the central amygdala of the “depressed” flinders sensitive line and the control flinders resistant line rats. Prog. Neuropsychopharmacol. Biol. Psychiatry 32, 651–661. 10.1016/j.pnpbp.2007.11.008 18077069

[B82] Zhu Y. Nachtrab G. Keyes P. C. Allen W. E. Luo L. Chen X. (2018). Dynamic salience processing in paraventricular thalamus gates associative learning. Science 362, 423–429. 10.1126/science.aat0481 30361366 PMC6521722

[B81] Zhu Y. Wienecke C. F. Nachtrab G. Chen X. (2016). A thalamic input to the nucleus accumbens mediates opiate dependence. Nature 530, 219–222. 10.1038/nature16954 26840481 PMC4814115

